# Supplementary material for: CryoEM structures of the multimeric secreted NS1, a major factor for dengue hemorrhagic fever
Source: Nat Commun. 2022 Nov 9;13:6756. doi: 10.1038/s41467-022-34415-1 (PMC9643530; doi:10.1038/s41467-022-34415-1)
Supplement: Supplementary file 1 — Supplementary Information [file 41467_2022_34415_MOESM1_ESM.pdf]

Supplementary information

CryoEM structures of the multimeric secreted NS1, a major factor for dengue hemorrhagic fever

Bo Shu, Justin S.G. Ooi, Aaron W.K Tan, Thiam-Seng Ng, Wanwisa Dejnirattisai, Juthathip Mongkolsapaya, Guntur Fibriansah, Jian Shi, Victor A. Kostyuchenko, Gavin R. Screaton, Shee-Mei Lok*

*Correspondence: Shee-Mei Lok (sheemei.lok@duke-nus.edu.sg)

Supplementary Fig. 1

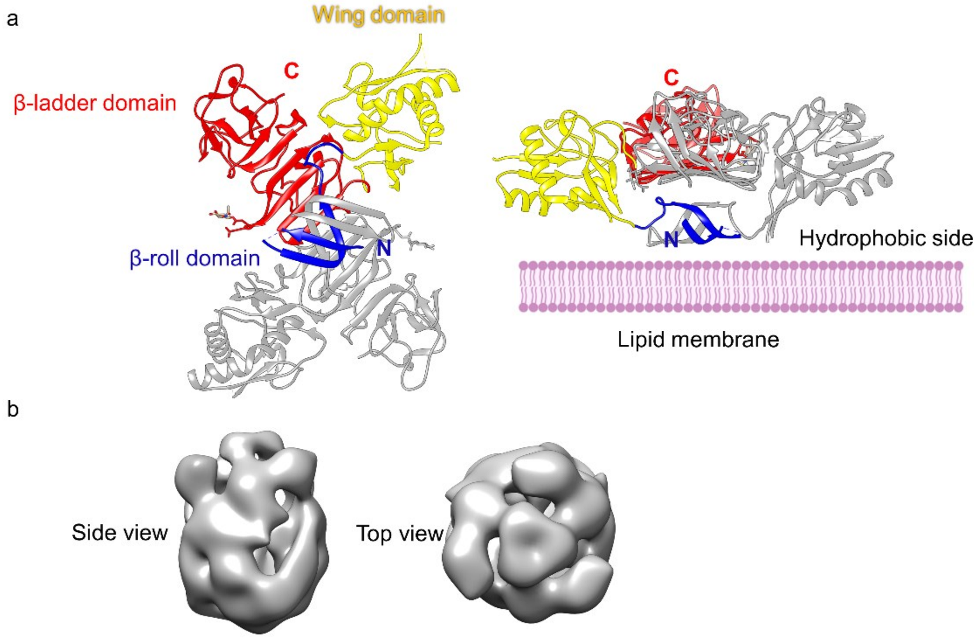

Supplementary Fig. 1 Previously published structures of the dengue NS1. (a) Crystal structure of dengue dimeric iNS1 (PDB:4O6B). Left: top view, looking from the β-roll of the iNS1 dimer. One protomer contains three domains: starting from the N-terminus- β-roll (blue), wing domain (yellow) and the β-ladder (red). It interacts with the other protomer (colored in grey) through extensive hydrogen bonds. Right: side view of the iNS1 dimer. The β-roll is highly hydrophobic and is predicted to interact with the cellular membrane. (b) A previously published low resolution EM reconstruction of the sNS1 (EMD-2073). This map is reconstructed from negatively stained sNS1 and C3 symmetry was imposed [14].

Supplementary Fig. 2

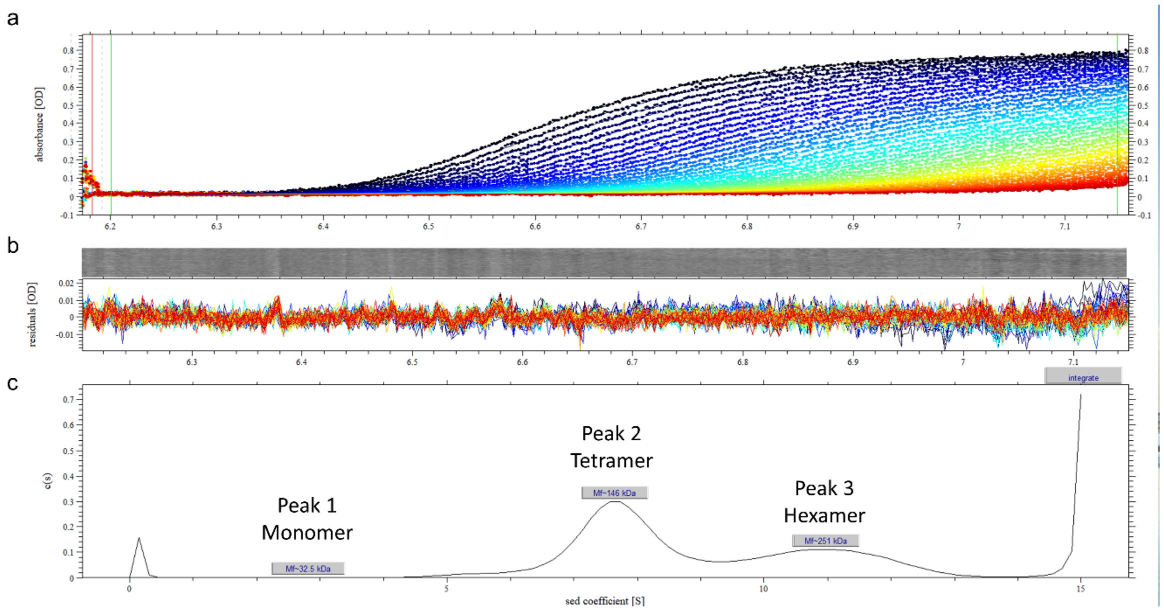

Supplementary Fig. 2 Sedimentation velocity analytical ultracentrifugation experiment of the recombinant sNS1. (a) Raw sedimentation profiles of absorbance at 280 nm versus particle radius for the recombinant sNS1. The sedimentation scans were coloured with the progressive rainbow colours according to the software default setting (b) Residual plot supplied by SEDFIT software showing the fitting goodness. (c) Continuous sedimentation coefficient distribution of the different sNS1 particle population – monomer, tetramers and hexamers with estimated MW of 32.5 kDa, 146 kDa and 251 kDa, respectively.

Supplementary Fig. 3

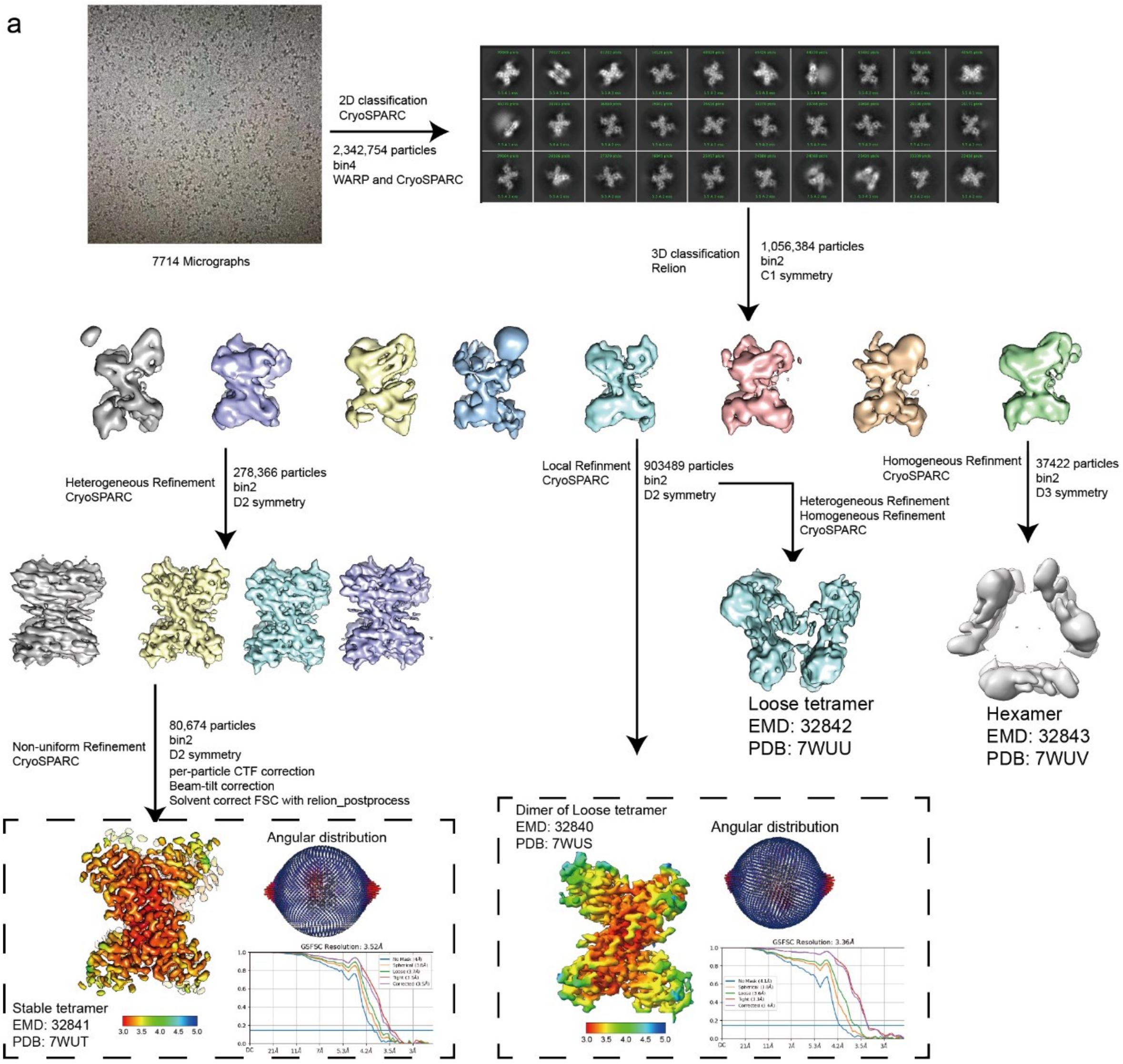

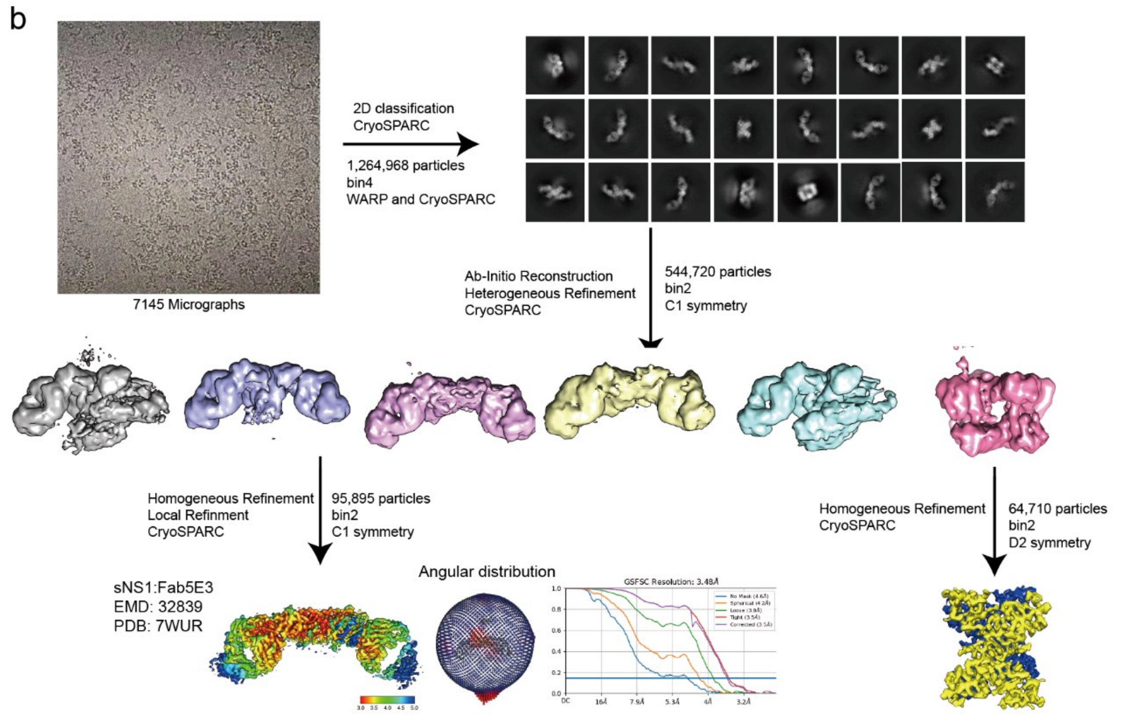

Supplementary Fig. 3 The cryoEM reconstruction workflow for uncomplexed sNS1 and NS1:Fab5E3 complex. (a,b) Representative micrograph, 2D class averages, representative 3D classes, map colored to resolution, and angular distribution of particles shown for cryoEM datasets.

Supplementary Fig. 4

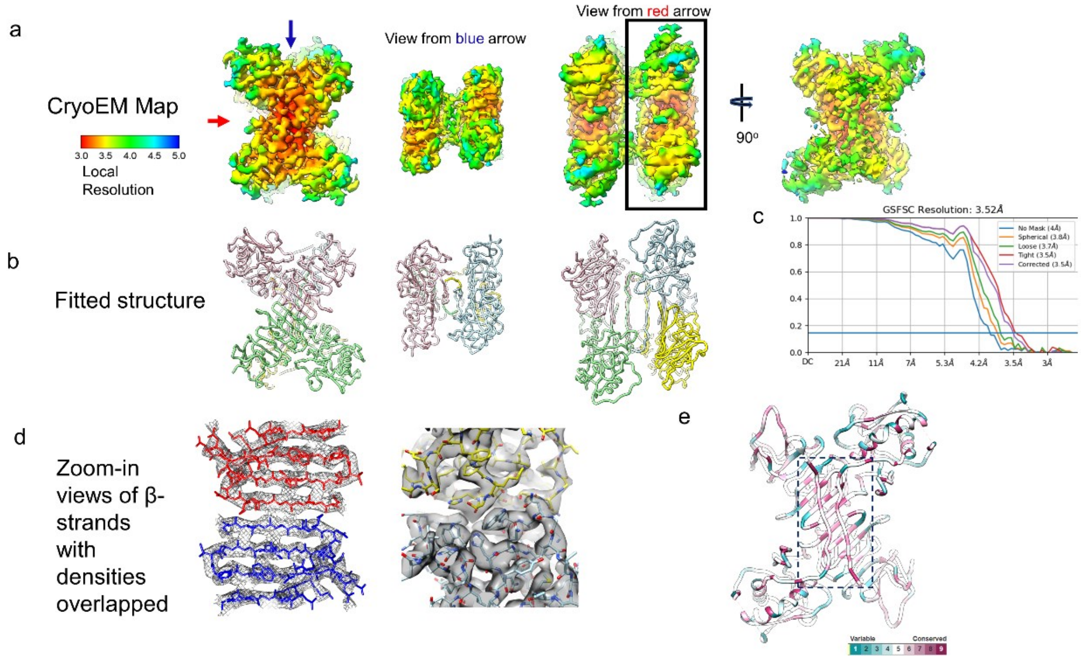

Supplementary Fig. 4 The 3.5Å resolution sNS1 stable tetrameric structure. (a) Different views of the cryoEM map displayed at high contour level. The densities are colored according to their local resolution. (b) The fitted structure show in the same view as the cryoEM map directly above (in (a)). There are two dimers facing each other. One dimer is colored in pink and green, while the other is light blue and yellow. (c) Fourier shell correlation plot showing resolution. (d) Zoom-in views of density map showing the β-strands densities are well resolved (left). Some of the side chains densities (right) can also be observed. (e) The NS1 surface is colored according to sequence conservation from the most conserved (dark magenta) to the most divergent (dark cyan) based on an alignment of NS1 sequences from 130 flaviviruses using the ConSurf server. The result shows that most region of the elongated β-sheet (dotted black lines) is conserved.

Supplementary Fig. 5

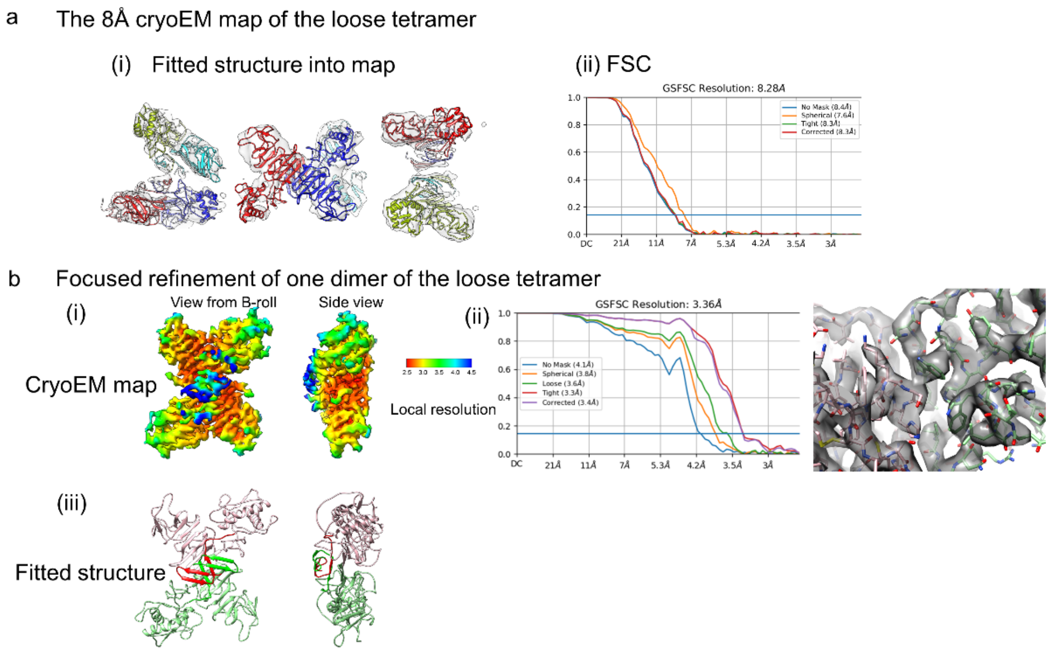

Supplementary Fig. 5 CryoEM maps of the sNS1 loose tetramer. (a) The 8Å cryoEM map of the loose tetramer. (i) Different views of the fitted structure of the loose tetramer into the density map (transparent grey). (ii) FSC plot showing resolution. (b) The 3.4Å cryoEM map of one dimer of the loose tetramer after focused refinement. (i) Different views of the cryoEM map. Density is colored according to local resolution. (ii) Up: FSC plot showing resolution of the map: Down: zoom-in view showing that some side chain densities are well resolved. (iii) The fitted structure of one of the dimers (pink and light green) is very similar to that of dengue iNS1. The β-roll in each protomer is colored in a darker shade of the respective protomer's color.

Supplementary Fig. 6

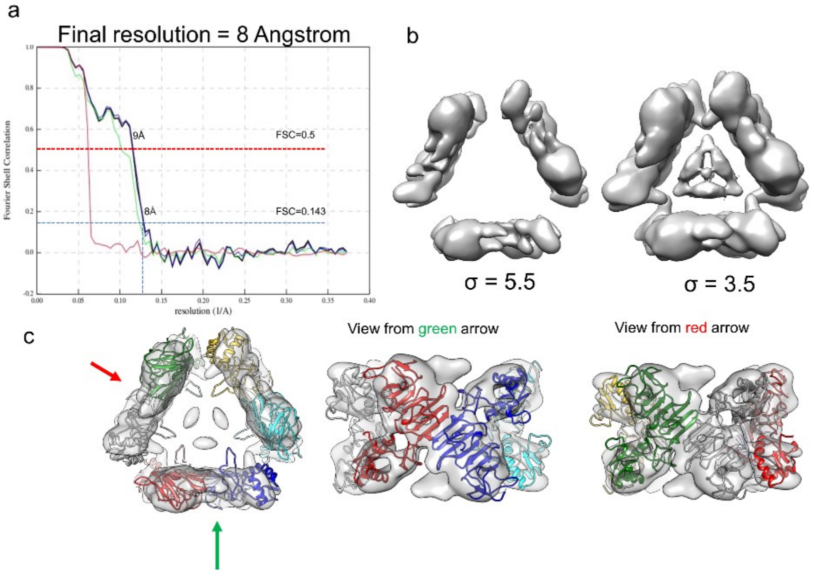

Supplementary Fig. 6 The 8Å cryoEM map of the sNS1 hexamer. (a) FSC plot showing resolution of the map. (b) The hexamer density displayed at different contour levels. At lower contour levels, densities at the interior of the hexamer can be seen. (c) Different views of the fit of the three dimers into the hexamer density map.

Supplementary Fig. 7

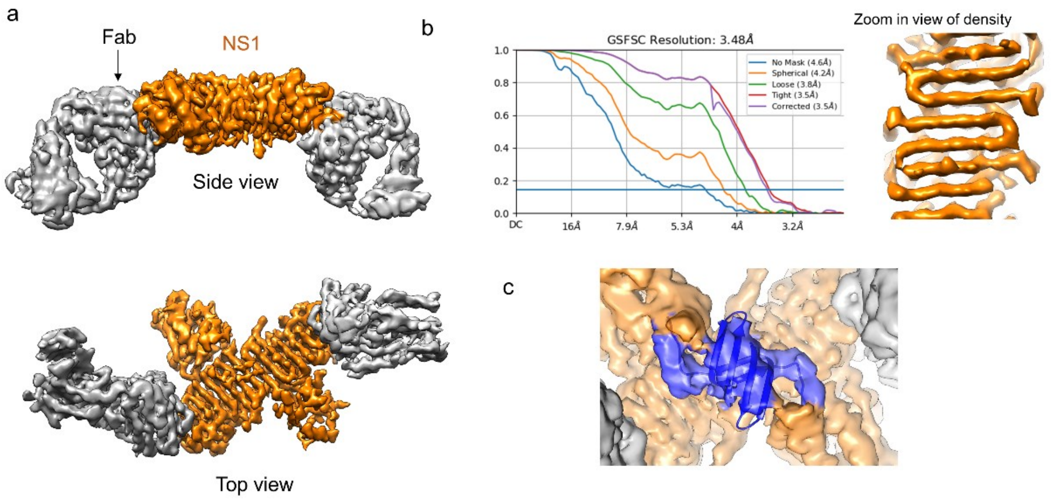

Supplementary Fig. 7 The 3.5Å resolution cryoEM structure of Fab 5E3:sNS1 dimer. (a) Side and top views of the cryoEM map of the Fab 5E3:sNS1 dimer. NS1 is colored in orange while Fabs are shown in grey. (b) Left: FSC plot showing the resolution of the map. Right: zoom-in view showing well-resolved β-strands. (c) Zoom-in view of the fitted β-roll into its corresponding density.

Supplementary Fig. 8

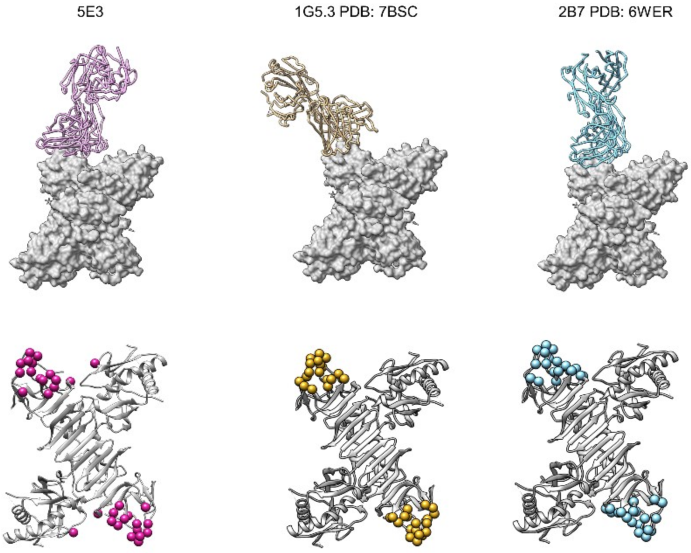

Supplementary Fig. 8 Comparison of the binding (top panels) and epitope (bottom panels) of Fab 5E3 to other previously published Fab:NS1 complexes [20,21]**.**

Supplementary Fig. 9

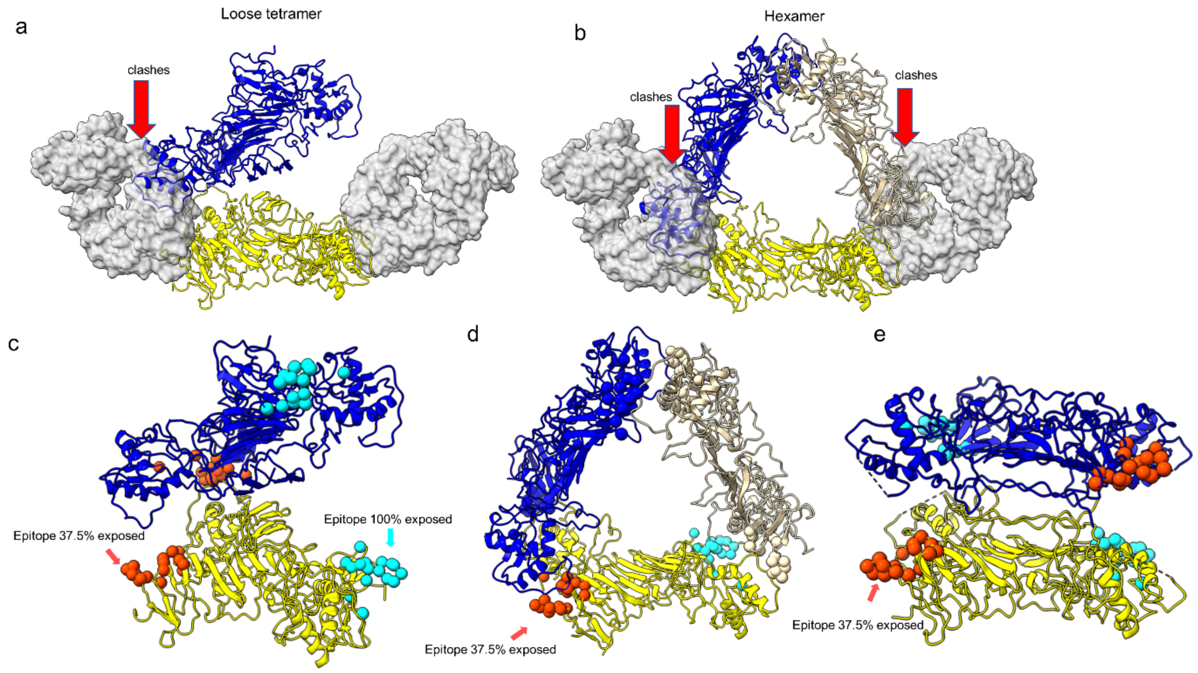

Supplementary Fig. 9 Superposition of Fab 5E3:dimer onto the (a) loose tetramer and (b) hexamer and the analysis of percentage exposure of epitopes (c-e) in the stable, loose tetramers and hexamers. (a-b) Superposition of Fab 5E3:dimer onto one dimer (yellow) of the loose tetramer and hexamer. Fab is shown as transparent grey surface and clashes with neighboring NS1 dimer are indicated with red arrows. (c-e) The percentage exposure of epitopes in the oligomers. The two epitopes within one dimer are colored in orange and cyan. (c) In the loose tetramer, 37.5% of the orange epitope is exposed while the cyan epitope is fully exposed (100%). (d) In the hexamer, 37.5% of both the orange and cyan epitopes are exposed. (e) In the stable tetramer, the two epitopes are both 37.5% exposed.

Supplementary Fig. 10

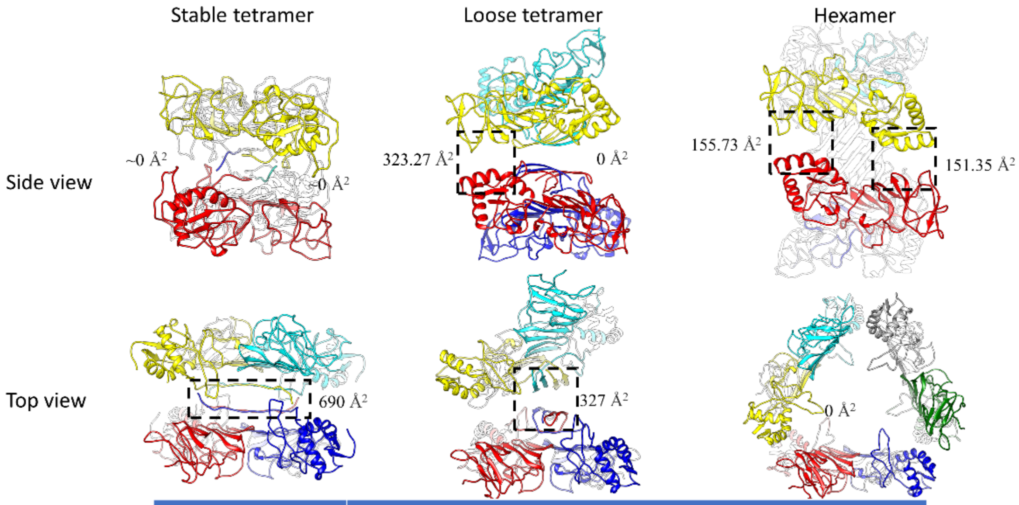

Oligomers	Buried surface(Å²)		
	Wing / β ladder		β-sheet or β-roll
Stable tetramer	0	0	690
Loose tetramer	323.27	0	327
Hexamer	155.73	151.35	0

Supplementary Fig. 10 Buried surface areas between two neighboring dimers of the different oligomeric states of the sNS1. The buried surface is calculated by the software ChimeraX.

Supplementary Fig. 11

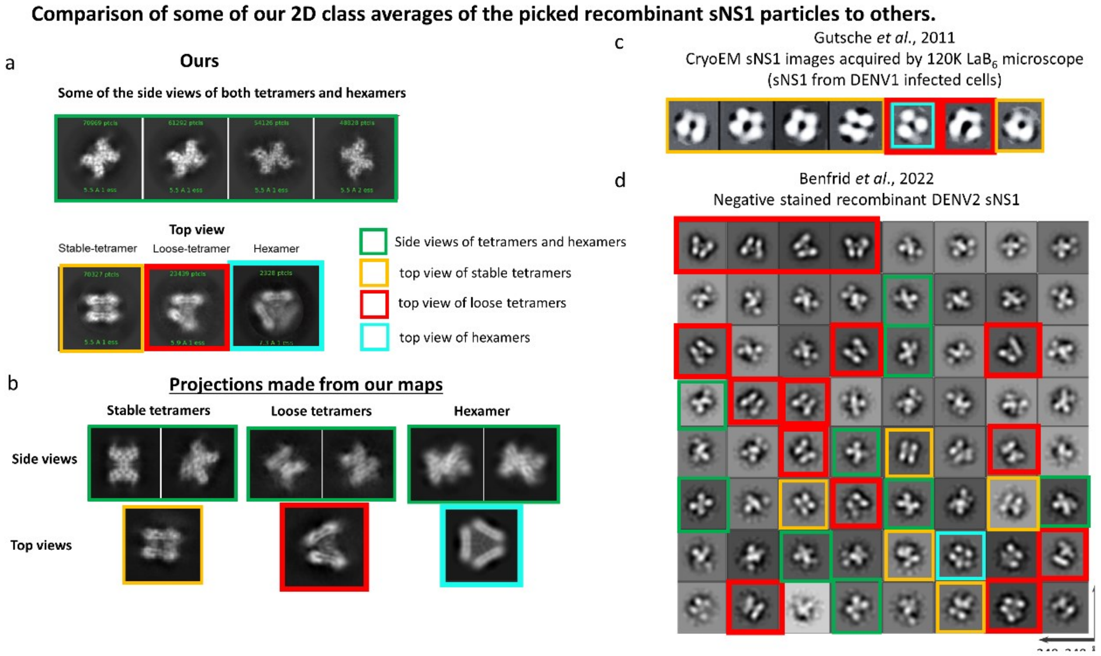

Supplementary Fig. 11 Comparison of the cryoEM micrographs and 2D averages from (a) our sample with that in (c) Gutsche et al., 2011[15] and (d) Benfrid et al., 2022[23]. (b) Projections made from our cryoEM maps of the stable and loose tetramers and also the hexamer representing some of the side and top views of each structure. Possible similar projections are boxed in the same color. In (d), the unboxed class averages are largely the different orientations of the side views. Panel c is obtained from Gutsche et al., 2011, while panel d, from Benfrid et al., 2022. We thank the corresponding authors and the journals, respectively, for the permission to use these figures.

Supplementary Fig. 12

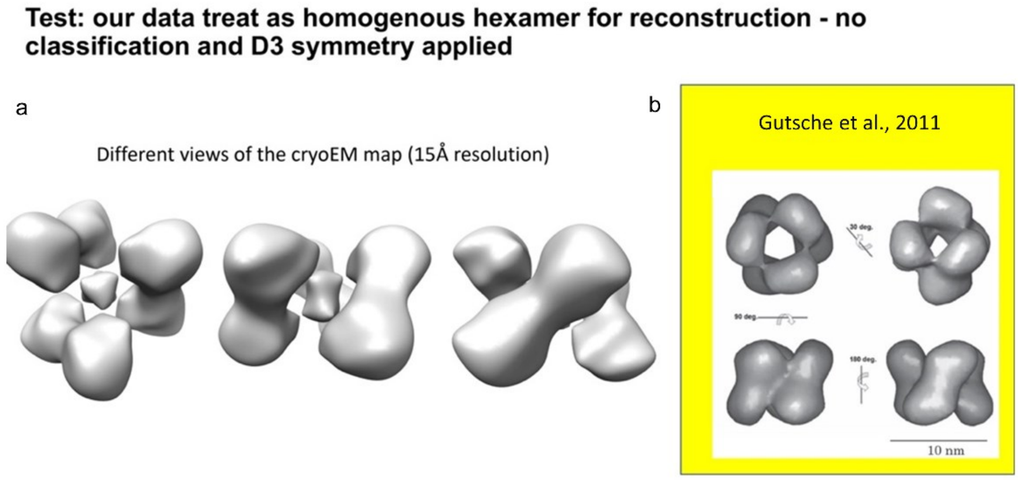

Supplementary Fig. 12 Comparison of the resultant cryoEM map of the test reconstruction with our dataset and assuming the particles are homogenously hexameric, with that obtained by Gutsche et al., 2011[15]. (a) Three dimensional reconstruction by using 30,000 randomly selected particles from our recombinant sNS1 sample and assuming the particles are homogenously hexamers (no classification was done and D3 symmetry was applied) shows a low resolution cryoEM map. This map appears to be similar to that reported by (b) Gutsche et al., 2011. This image is obtained from Gutsche et al., 2011, we thank the corresponding author for the permission to use it.

Supplementary Fig. 13

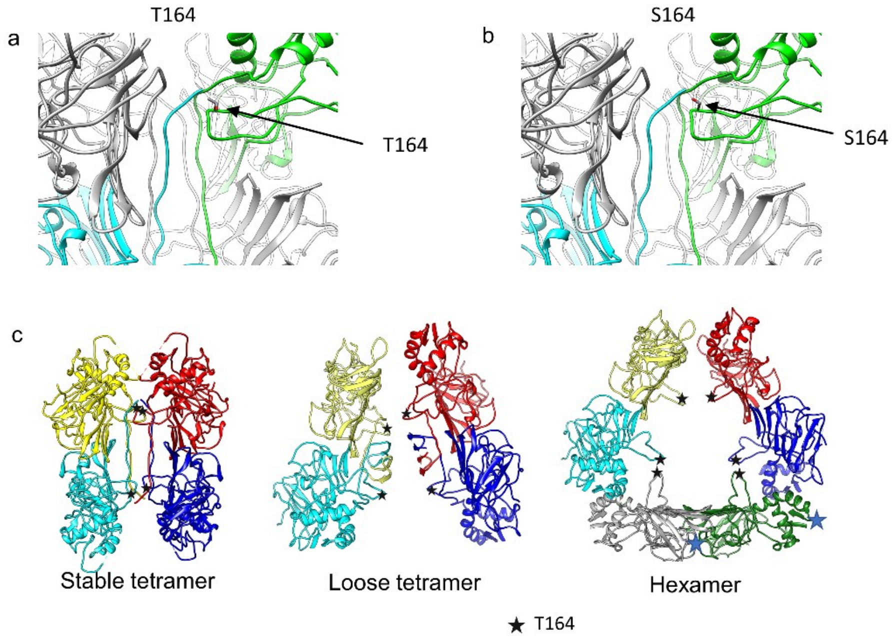

Supplementary Fig. 13 Zoom-in view of regions around residue 164. (a) Zoom-in view of regions around residue 164. This residue lies in close proximity (Cα-Cα backbone distance is 5.8A) to the highly hydrophilic elongated β-sheet of the opposite dimer. (b) A homology model of stable tetramer with S164 mutation showed that the mutation might increase the hydrophilicity of that region leading to increased interactions with the opposite dimer. (c) black Five-pointed star indicates where the T164 are in different oligomers of the sNS1

	DENV2_NS1_Tetramer	DENV2_NS1_dimer	DENV_NS1_Fab5E3
**Data collection and processing**	(EMDB-32841)<br>(PDB 7WUT)	(EMDB-32840)<br>(PDB 7WUS)	(EMDB-32839)<br>(PDB 7WUR)
Microscope	FEI Titan Krios	FEI Titan Krios	FEI Titan Krios
Camera	Gatan K3	Gatan K3	Gatan K3
Magnification	130,000 ×	130,000 ×	130,000 ×
Voltage (kV)	300	300	300
Electron exposure (e^-/Å^2)	81.5	81.5	80.1
Defocus range (μm)	-0.8 to -2.0	-0.8 to -2.0	-0.8 to -2.0
Exposure rate (e^-/A^2/sec)	8.74	8.86	8.35
Number of frames per movie	58	58	58
Pixel size (Å)	0.66	0.66	0.66
Energy filter slit width (eV)	20	20	20
Automation software	SerialEM	SerialEM	SerialEM
Symmetry imposed	D2	C1	C1
Micrographs used	7,714	7,714	7,145
Total number of extracted particles	2,342,754	2,342,754	1,264,968
Total number of refined particles	278,366	454,477	273,074
Number of particles in final map	80,674	181,587	95,895
Map resolution (Å)	3.5	3.3	3.5
FSC threshold	0.143	0.143	0.143
**Refinement**			
Initial model used (PDB code)	5GS6	5GS6	5GS6
Refinement package, (resolution cutoff)	Phenix, real-space refinement (3.7)	Phenix, real-space refinement (3.4)	Phenix, real-space refinement (3.5)

Resolution of unmasked and masked reconstructions at 0.5 and 0.143 FSC	Unmasked<br>3.5/4.1<br>Masked<br>3.4/4.1	Unmasked<br>3.6/4.0<br>Masked<br>3.5/3.9	Unmasked<br>3.4/3.8<br>Masked<br>3.4/3.6
Map sharpening *B* factor (Å^2)	-230	-210	-105.9
Model composition			
Non-hydrogen atoms	9,732	5,450	8,922
Protein residues	1264	682	1134
Ligands	0	0	0
Global CC (CCvol)	0.77	0.74	0.84
Local CC (CCmask)	0.78	0.75	0.84
B factors (Å^2)			
Protein	92.57	52.17	89.33
Ligand	-	-	-
R.m.s. deviations			
Bond lengths (Å)	0.003	0.003	0.003
Bond angles (°)	0.834	0.753	0.666
Validation			
MolProbity score	2.29	2.10	2.04
Clashscore	13.93	11.9	11.28
Poor rotamers (%)	0.20	0.67	0.31
C-beta deviations	0.00	0.00	0.00
Ramachandran plot			
Favored (%)	86.38	91.45	92.42
Allowed (%)	13.30	8.55	7.58
Disallowed (%)	0.32	0	0
CaBLAM outliers (%)	7.63	5.93	3.78

EMRinger score	1.34	1.76	2.51

Supplementary Table 1 Cryo-EM data collection, refinement and validation statistics

a

	Stable Tetramer	Loose Tetramer	Hexamer
Particles	126130	90928	0
Percentage	58%	42%	0 %

b

	Stable Tetramer	Loose Tetramer	Hexamer
Particles	315671	102939	0
Percentage	75.4%	24.6%	0 %

Supplementary Table 2 The percentage of particles in different oligomerization states determined after 3D classification of (a) sNS1 treated with detergent (0.05% DDM) and (b) the STconc_sNS1 sample.

NS1	Fab Heavy Chain	Fab Light Chain
138		27(CDR1)
269		94(CDR3)
281	52/53/54/55/56(CDR2)	
299	102/103(CDR3)	
301	33(CDR1)	
302	103(CDR3)	
303	103(CDR3)	
304	32/106(CDR1/CDR3)	
305	31/33/103(CDR1/CDR3)	
306	31(CDR1)	
307	31/53(CDR1/CDR2)	
324	56(CDR2)	
325	57(CDR2)	
326	57/102/100(CDR2/CDR3)	
327	100/102(CDR3)	94(CDR3)
328	100(CDR3)	

Supplementary Table 3 List of hydrogen bonds/electrostatic interactions between Fab 5E3 (heavy and light chains) with the NS1 protein. The interacting CDRs of the Fabs are indicated.
