# Supplementary material for: CryoEM structures of the multimeric secreted NS1, a major factor for dengue hemorrhagic fever
Source: Nat Commun. 2022 Nov 9;13:6756. doi: 10.1038/s41467-022-34415-1 (PMC9643530; doi:10.1038/s41467-022-34415-1)
Supplement: Supplementary file 3 — Description of Additional Supplementary Files [file 41467_2022_34415_MOESM3_ESM.pdf]

Description of Additional Supplementary Files

File Name: Supplementary Movie 1
Description: Structure of the stable sNS1 tetramer and the zoom-in view of its elongated β-sheet. Two NS1 dimeric structures fitted into the cryoEM map. Protomers within one dimer is colored in pink and light green while that in the opposite dimer, in yellow and light blue and their respective elongated β-strands are highlighted in darker shades - red, dark green, brown and blue, respectively.
