# Supplementary material for: CryoEM structures of the multimeric secreted NS1, a major factor for dengue hemorrhagic fever
Source: Nat Commun. 2022 Nov 9;13:6756. doi: 10.1038/s41467-022-34415-1 (PMC9643530; doi:10.1038/s41467-022-34415-1)
Supplement: Supplementary file 5 — Reporting Summary [file 41467_2022_34415_MOESM5_ESM.pdf]

nature portfolio

Corresponding author(s): SML
Last updated by author(s): May 30, 2022

Reporting Summary

Nature Portfolio wishes to improve the reproducibility of the work that we publish. This form provides structure for consistency and transparency in reporting. For further information on Nature Portfolio policies, see our Editorial Policies and the Editorial Policy Checklist.

Statistics

For all statistical analyses, confirm that the following items are present in the figure legend, table legend, main text, or Methods section.

n/a	Confirmed	
☐	☒	The exact sample size (*n*) for each experimental group/condition, given as a discrete number and unit of measurement
☐	☒	A statement on whether measurements were taken from distinct samples or whether the same sample was measured repeatedly
☐	☒	The statistical test(s) used AND whether they are one- or two-sided<br>*Only common tests should be described solely by name; describe more complex techniques in the Methods section.*
☒	☐	A description of all covariates tested
☐	☒	A description of any assumptions or corrections, such as tests of normality and adjustment for multiple comparisons
☐	☒	A full description of the statistical parameters including central tendency (e.g. means) or other basic estimates (e.g. regression coefficient) AND variation (e.g. standard deviation) or associated estimates of uncertainty (e.g. confidence intervals)
☐	☒	For null hypothesis testing, the test statistic (e.g. *F*, *t*, *r*) with confidence intervals, effect sizes, degrees of freedom and *P* value noted<br>*Give P values as exact values whenever suitable.*
☒	☐	For Bayesian analysis, information on the choice of priors and Markov chain Monte Carlo settings
☒	☐	For hierarchical and complex designs, identification of the appropriate level for tests and full reporting of outcomes
☒	☐	Estimates of effect sizes (e.g. Cohen's *d*, Pearson's *r*), indicating how they were calculated

Our web collection on statistics for biologists contains articles on many of the points above.

Software and code

Policy information about availability of computer code

Data collection	For cryoEM data accquision and processing: SerialEM 3.8, MotionCorr2, CTFFIND4.1.13, Relion3.1, Cryosparc3.0.1,Phenix-1.18.2 and coot0.8.2
Data analysis	For model building and analysis: UCSF-Chimera1.13.1<br>For structural figure: UCSF-Chimera1.13.1; UCSF-ChimeraX1.1<br>For data processing: Graphpad-9.2.0<br>For figures: Adobe illustratror(CS6)

For manuscripts utilizing custom algorithms or software that are central to the research but not yet described in published literature, software must be made available to editors and reviewers. We strongly encourage code deposition in a community repository (e.g. GitHub). See the Nature Portfolio guidelines for submitting code & software for further information.

Data

Policy information about availability of data

All manuscripts must include a data availability statement. This statement should provide the following information, where applicable:

- Accession codes, unique identifiers, or web links for publicly available datasets
- A description of any restrictions on data availability
- For clinical datasets or third party data, please ensure that the statement adheres to our policy

The cryoEM map and the atomic models of the all sNS1 and sNS1:Fab structures have been deposited in the Electron Microscopy Data Bank and the Protein Data Bank - stable tetramer (EMD: 32841, PDB: 7WUT), loose tetramer (EMD: 32842 and PDB: 7WUU), Dimer of Loose tetramer (EMD: 32840 and PDB: 7WUS), hexamer (EMD: 32843 and PDB: 7WUV) and sNS1:Fab5E3(EMD: 32839 and PDB: 7WUR). The previously published NS1 structures used in this study is available in the Protein Data Bank under accession codes 5GS6 and 4O6B.

Field-specific reporting

Please select the one below that is the best fit for your research. If you are not sure, read the appropriate sections before making your selection.

☒ Life sciences ☐ Behavioural & social sciences ☐ Ecological, evolutionary & environmental sciences

For a reference copy of the document with all sections, see nature.com/documents/nr-reporting-summary-flat.pdf

Life sciences study design

All studies must disclose on these points even when the disclosure is negative.

Sample size	For cryoEM analysis, 7714 images were used for NS1 structure reconstruction and 7145 images for NS1-Fab5E3 reconstruction.
Data exclusions	1020 images of NS1 and 242 images of NS1-Fab5E3 were excluded owing to the bad qualities.
Replication	For NS1 dataset, we collected three datasets, and the results keep consistent. And the NS1-Fab5E3 dataset were collected from one cryo grid.
Randomization	During 3D auto-refinement for CryoEM analysis, each dataset was randomly split to two half for calculating the Fourier shell correlation(FSC)
Blinding	Blinding was not feasible to this study. since we are investigating a protein structure, there was no blinding in data collection and structural analysis.

Reporting for specific materials, systems and methods

We require information from authors about some types of materials, experimental systems and methods used in many studies. Here, indicate whether each material, system or method listed is relevant to your study. If you are not sure if a list item applies to your research, read the appropriate section before selecting a response.

Materials & experimental systems

n/a	Involved in the study	
☐	☒	Antibodies
☐	☒	Eukaryotic cell lines
☒	☐	Palaeontology and archaeology
☒	☐	Animals and other organisms
☒	☐	Human research participants
☒	☐	Clinical data
☒	☐	Dual use research of concern

Methods

n/a	Involved in the study	
☒	☐	ChIP-seq
☒	☐	Flow cytometry
☒	☐	MRI-based neuroimaging

Antibodies

Antibodies used	Fab_5E3 was isolated and purified by Gavin Screaton's lab. Anti polyHistidine–peroxidase antibody was bought from Sigma-Aldrich (cat: A7058-1VL). It was used for western blot with a dilution of 1: 1000.
Validation	Antibody 5E3 was produced in Gavin Screation's lab, it was validated for binding to recombinant, purified NS1 by ELISA, Octet and CryoEM analysis.<br>Anti polyHistidine–peroxidase antibody: https://www.sigmaaldrich.com/SG/en/product/sigma/a7058?gclid=CjwKCAjw-rOaBhA9EiwAUkLV4nxR2IzFOEI2hKWl6F5IfvKGqeJfYg7Oiljjk6BRvP4ddkkKWBP2iBoCRtIQAvD_BwE

Eukaryotic cell lines

Policy information about cell lines

Cell line source(s)	Expi293F cells and Aedes albopictus clone C6/36
Authentication	The Expi293F cell was purchased and authenticated by from Thermo Fisher(cat number:A14635).<br>Aedes albopictus clone C6/36 cell was purchased and authenticated by from ATCC.
Mycoplasma contamination	original ampules purchased were certified free of mycoplasma by company
Commonly misidentified lines (See ICLAC register)	Not listed in the commonly misidentified lines.
